# A novel 9-bp insertion detected in steroid 21-hydroxylase gene (CYP21A2): prediction of its structural and functional implications by computational methods

**DOI:** 10.1186/1423-0127-16-3

**Published:** 2009-01-08

**Authors:** Sudhisha Dubey, Susan Idicula-Thomas, Mohammad Anwaruddin, Chinnaraj Saravanan, R Raveendra Varma, Anurupa Maitra

**Affiliations:** 1 Department of Molecular Endocrinology, National Institute for Research in Reproductive Health, Indian Council of Medical Research, J M Street, Parel, Mumbai, Maharashtra, India; 2 Biomedical Informatics Centre of Indian Council of Medical Research, National Institute for Research in Reproductive Health, J M Street, Parel, Mumbai, Maharashtra, India; 3 Department of Pediatrics and Neonatology, Mother's Hospital Trissur, Kerala, India

## Abstract

**Background:**

Steroid 21-hydroxylase deficiency is the most common cause of congenital adrenal hyperplasia (CAH). Detection of underlying mutations in *CYP21A2 *gene encoding steroid 21-hydroxylase enzyme is helpful both for confirmation of diagnosis and management of CAH patients. Here we report a novel 9-bp insertion in *CYP21A2 *gene and its structural and functional consequences on P450c21 protein by molecular modeling and molecular dynamics simulations methods.

**Methods:**

A 30-day-old child was referred to our laboratory for molecular diagnosis of CAH. Sequencing of the entire *CYP21A2 *gene revealed a novel insertion (duplication) of 9-bp in exon 2 of one allele and a well-known mutation I172N in exon 4 of other allele. Molecular modeling and simulation studies were carried out to understand the plausible structural and functional implications caused by the novel mutation.

**Results:**

Insertion of the nine bases in exon 2 resulted in addition of three valine residues at codon 71 of the P450c21 protein. Molecular dynamics simulations revealed that the mutant exhibits a faster unfolding kinetics and an overall destabilization of the structure due to the triple valine insertion was also observed.

**Conclusion:**

The novel 9-bp insertion in exon 2 of *CYP21A2 *genesignificantly lowers the structural stability of P450c21 thereby leading to the probable loss of its function.

## Background

Congenital adrenal hyperplasia (CAH; OMIM# 201910) is an autosomal recessive disorder caused by deficiency of one of the five steroidogenic enzymes involved in cortisol biosynthesis. Steroid 21-hydroxylase deficiency accounts for about 90–95% of all CAH cases [1]. Deficiency of cortisol results in excessive production of androgens leading to prenatal virilization in females and rapid somatic growth in both sexes [2]. CAH has been traditionally divided into three forms; severe salt wasting (SW), less severe simple virilizing (SV) and asymptomatic non-classic (NC) form. The SW form is common and found in 75% of all CAH patients. In addition to decreased cortisol, aldosterone biosynthesis is also impaired in these patients resulting in severe renal salt loss and hypotonic shock, unless treated during the neonatal period [2]. Patients with SV form do not have aldosterone deficiency and thus salt loss is not present. Symptoms like variable degree of ambiguous genitalia in females, growth acceleration and pseudoprecocious puberty are seen in SV forms. The milder non-classical form is asymptomatic at birth and presents with various degrees of late onset features of hyperandrogenism.

The wide spectrum of clinical manifestations seen in this disorder is due to varied degrees of enzyme activity caused by different mutations in the *CYP21A2 *gene encoding the steroid 21-hydroxylase enzyme. Although, both large and point mutations have been seen in different populations [3-7], point mutations constitute a larger proportion. The *CYP21A2 *gene is part of a complicated structure, referred to as the RCCX-module located in human leukocyte antigen class III locus on chromosome 6p21.3 [8]. Approximately 30 kb upstream of *CYP21A2*, a pseudogene (*CYP21A1P*) is present; which is 98% identical to *CYP21A2. CYP21A1P *cannot synthesize the functional protein due to presence of numerous deleterious mutations.

About 95% of the mutated alleles in patients with steroid 21-hydroxylase deficiency are generated by transfer of DNA sequences from *CYP21A1P *to *CYP21A2 *by gene conversion events [9]. The remaining 5% alleles have new/rare mutations due to random events [2]. The number of rare mutations identified has increased dramatically in the last few years [10]. Most of these mutations are unique to individual families but some are population specific [11-13]. Functional characterization of most of the mutants using site directed mutagenesis followed by *in vitro *expression analyses have been helpful in correlating clinical severity to the degree of loss of enzyme function caused by the mutations [10]. However such studies are difficult to perform in routine laboratory set up with limited resources. Alternatively, these experimental assays could be complemented with computational studies; wherein the structural and functional perturbations of a protein by virtue of mutation/s could be predicted. Consequence of single mutation (W62G) on structural stability of lysozyme [14] and structural perturbations caused by three different activating mutations of the *hLHR *gene found in four unrelated Brazilian boys with male-limited precocious puberty have been studied by MD simulations [15]. Computational scientists have also used MD simulations to independently explore the role of mutations on protein stability and activity [16-22]. In the present study, molecular modeling and MD simulations were carried out to understand the structural and probable functional implications of the triple valine insertion of human P450c21.

## Materials and methods

### Clinical history of proband

The proband presented on day 30 with ambiguous genitalia and failure to thrive. She was the first child born of non-consanguineous marriage with an uneventful antenatal history. She was born at full term by normal delivery. Her clinical examination showed prominent phallus with clitoral hypertrophy and partial fusion of labia (Prader stage IV). Gonads were non-palpable. Genitogram and panendoscopy revealed normal bladder, common urogenital sinus, vagina, and uterine cervix. Her weight was initially 2.9 kg (well below the 0.4^th ^centile). Later at 12 weeks her length was 54.7 cm (2^nd ^centile) and weight 4.3 kg (2^nd ^centile). Hormonal examination revealed elevated 17-hydroxyprogesterone (17-OHP) (8800 ng/dl; normal value < 630 ng/dl), testosterone (1.5 ng/ml; normal range 0–0.28 ng/ml) and low cortisol (5.6 ug/dl; normal range 11–18 ug/dl). Sodium and potassium levels were 138 mmol/l and 6.1 mmol/l respectively. Her chromosomal analysis revealed 46, XX normal karyotype. She was diagnosed as simple virilising CAH and put on a relatively low dose of 5 mg/m^2 ^of hydrocortisone and 50 μg of fludrocortisone daily. In second year, she showed normal development with 17-OHP level of 560 ng/dl. Her weight was 8 kg, height – 74 cm and head circumference – 45.3 cm (all below 0.4^th ^centile). Her bone age was normal. She was continued with the same dose till her third year. Thereafter her dose had to be increased to 9 mg of hydrocortisone and 100 μg of fludrocortisone daily due to increased level of 17-OHP (7200 ng/dl). She is now 5 yrs old and developing normally.

### Molecular analysis

Informed consent was obtained from both parents and the study was approved by the Ethical Committee of our institution. About 2 ml of whole blood was collected in EDTA vacutainers (Becton-Dickinson, USA) for *CYP21A2 *gene analysis and DNA was extracted using Qiagen kit (QIAmp DNA Blood Kit, QIAGEN GmbH, Hilden). 200 ng of genomic DNA was subjected to selective amplification of *CYP21A2 *gene in two different fragments of 1.2 and 2.3 kb respectively using previously described primers [23]. PCR was carried out in 2720 thermocycler (Applied Biosystem) in a reaction volume of 50 μl containing 1.5 mM MgCl_2_, 0.2 mM dNTPs, 200 mM (NH4)_2_SO_4_, 750 mM Tris-HCl (pH 8.8), 0.1% Tween 20, 2 units of Taq polymerase (Fermentas, Life Sciences) and 0.5 μM of primers. PCR was performed at 96°C for 3 min for initial denaturation followed by 30 cycles of 95°C for 1 min, 55.5°C for 25 sec, and 72°C for 3 min and final extension at 72°C for 6 min. PCR products were resolved on 1% agarose gel stained with ethidium bromide, visualized under UV transilluminator.

The PCR products were purified using QIAGEN kit (QIAmp Gel Purification Kit, QIAGEN GmbH, Hilden). Purified products were subjected to direct sequencing with ten different primers (Table 1) based on dideoxynucleotide terminator methodology using the BigDye Terminator Cycle Sequencing Ready Reaction kit (PE, Applied Biosystems, Perkin Elmer Corp., Foster City, CA). The analysis was carried out in the ABI PRISM 3130 Genetic Analyser (PE, Applied Biosystems). The numbering of the nucleotides was according to Higashi et al [24].

**Table 1:** List of primers used for sequencing the entire *CYP21A2*

***Primer***	***Primer sequence***	***Nucleotide Number***	***Size***	***Location***
1F	5' aca gtc tac aca gca gga gg3'	-78–(-59)	20	Promoter
1R	5' gtg agg gcc aga gcg aga t 3'	214–232	19	Intron 1
2F	5' gag gac cat tga tga agc 3'	322–339	18	Exon 2
2R	5' ctc aca gaa ctc ctg ggt ca 3'	806–825	20	Exon 3
4F	5' cct gtc ctt ggg aga cta ct 3'	700–719	20	Exon 3
4R	5' gtc cac aat ttg gat gga cca 3'	1187–1207	21	Exon 5
6R	5' agc aat gct gag gcc ggt ag 3'	1460–1479	20	Intron 6
7F	5' att gct atg agg cgg gtt c3'	1474–1492	19	Intron 6
8F	5' ctc act ggg ttg ctg agg gag 3'	1901–1921	21	Intron 7
9F	5' ctt cag cat ctc cgg cta c 3'	2238–2256	19	Intron 8
10R	5' ctg tgt tta cag ggg gga 3'	2778-2796	18	Intron 10

### Web-based tools

The web-based tools were used for the purpose of secondary structure prediction, template recognition, model evaluation and sequence analysis (Table 2).

**Table 2:** Description of the web-based tools used in the study

**Tool**	**Description**	**URL**	**Reference**
WebLogo	Sequence Logo		41
FUGUE	Template identification		28
JPRED	Secondary structure prediction		26
GeneSilico metaserver	Fold prediction metaserver		25
SYMPRED	Secondary structure prediction		27
Verify3D	Structure evaluation		30
COLORADO3D	Structure evaluation		31
PSI-BLAST	Homolog identification		42

### Secondary structure prediction

GeneSilico metaserver, JPRED and SYMPRED servers [25-27] were used to predict the secondary structures of human P450c21 (Table 2). The output of each of these secondary structure prediction servers is the consensus secondary structure predicted for the protein based on a collection of independent algorithms. The SYMPRED result is based on the consensus formed by the predictions of HD, PROF, SSPRO, YASPIN, JNET and PSIPRED programs while GeneSilico result is based on PSIPRED and JNET.

### Identification of suitable template

FUGUE (v2.s.07; Table 2) was used for identification of template for modeling the structure of human P450c21 [28]. The best template is identified based on their Z-scores. The templates which have a Z-score of 6 or more are labeled as "*certain*" with 99% confidence.

### Assessment of the theoretical structure available for human P450c21

The theoretical structure of human P450c21 (PDB ID: 2GEG) has been elucidated using rabbit P4502c5 (PDB ID: 1N6B) as template [29]. The quality of this theoretical structure was assessed in two ways. Firstly, using the alignment of the target-template provided by FUGUE, the agreement between the predicted consensus secondary structure of human P450c21 with that of rabbit P4502c5 secondary structure was compared. Secondly, the quality of the theoretical model was evaluated using Verify3D [30] and Colorado3D server [31] (Table 2).

### Generation of structure for the mutant

The structure of mutant (MT) was modeled using the theoretical structure of wild-type (WT; 2GEG) as the template. Discovery Studio v 1.7 (Accelrys) was used for building the homology-based model.

### MD simulations

MD simulations were performed using GROMACS 3.3.1 [32]. United atom representation was used except for polar and aromatic ring hydrogen atoms. GROMOS96 forcefield was used for energy calculations. Van der Waals interactions were calculated with a distance cut-off of 0.9 nm. Electrostatic interactions were treated using the cut-off method [33]. Neutralizing counter ions were added when charged residues were present. The models were solvated with SPC water molecules [34] and simulated in a triclinic box [35,36] with periodic boundary conditions. The simulations were performed in the canonical NVT ensemble. The models were first energy minimized using steepest descent algorithm with a tolerance of 1 J mol^-1 ^nm^-1^; this was followed by position restrained MD simulations for 10 ps. Initial velocities were generated conforming to Maxwell velocity distribution. A time step of integration of 2 fs was used. LINCS algorithm was used to constrain the bonds [37]. The productive run was initiated for 10 ns; this duration was sufficient to compare the stabilities of the protein structures. The simulations were performed at 300K.

### Analysis of MD trajectories

With an objective of understanding the structural and functional implications of the triple valine insertion, trajectories of WT and MT were analyzed for the following structural properties as a function of time: a) Potential energy, b) The root mean square deviation (RMSD) of the Cα atoms with respect to the starting conformation, c) RMSD of the hydrophobic residues postulated to be involved in membrane binding [29] with respect to the starting conformation d) Distance between the Cα atoms of residues S108 and D287; both of which are suggested to be involved in heme and substrate-binding [29], e) Structural perturbations near the insertion site. The trajectories of the simulations were plotted using XMGRACE [38]. DSSP [39] program was used for secondary structure assignment and MolScript [40] was used to create and render the molecular images.

### Sequence logo

For understanding the conservation of residues in the immediate vicinity of the insertion, a sequence logo was constructed using the program WebLogo [41]. Proteins that share more than 30% similarity with human P450c21 and are deposited in the SwissProt database were identified using the PSI-BLAST algorithm [42]. The 50 identified homologs were all cytochromes and shared similarity throughout the length of the protein. These proteins were then subjected to multiple sequence alignment using CLUSTALW [43] and a sequence logo was constructed.

## Results

### Mutational analysis

**Figure 1:**
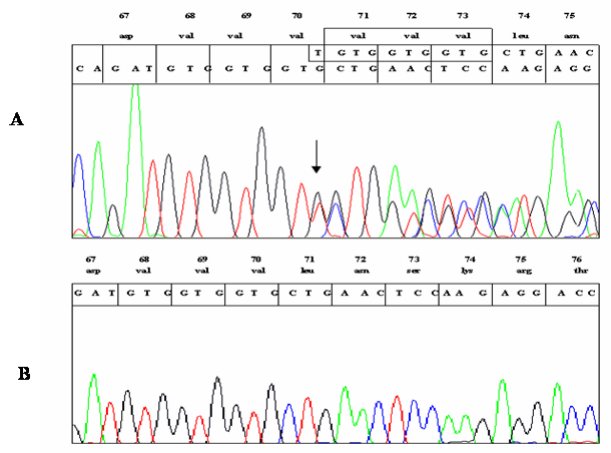
**Direct DNA sequencing of exon 2 of CYP21A2 gene**. (A) Sequence of mutated allele showing heterozygous insertion in exon 2 resulting in additional three valine residues after codon 70. The inserted sequence and the corresponding valine residues are boxed. The site of insertion is indicated by an arrow. (B) The lower sequence corresponds to the wild-type allele showing three valine residues.

Clinical and biochemical findings in our patient were consistent with her simple virilizing phenotype. Entire sequencing of *CYP21A2 *gene (including 10 exons, 9 introns and < 400 base pairs upstream to the transcription initiation codon) was performed in order to detect known and unknown mutations. Electropherogram showed a known I172N mutation in exon 4 of one allele and an in-frame insertion of 9-bp TGTGGTGGT at nucleotide 306 in exon 2 of other allele (Fig. 1A). This 9-bp insertion resulted in triple valine insertion between V70 and L71 of the wild type P450c21 (Fig. 1B). Re-sequencing of Exon 2 using reverse primer 2R (Table 1) showed a frameshift 9-bp away from the frameshift seen in the forward strand. Using SeqScape v2.1.1, forward and reverse sequences were aligned against the reference sequence (see Additional file 1 part A) that confirmed the insertion as highlighted sequences (see Additional file 1 part B). The shift in the insertion site was clearly observed in the forward (see Additional file 1 part C) and reverse (see Additional file 1 part D) strand that further confirmed this insertion as duplication of TGTGGTGGT base pairs in exon 2, at nucleotide position 306 of *CYP21A2 *gene (see Additional file 1 part E). This novel insertion mutation was inherited from the father, while the I172N was inherited from the mother, but both parents were heterozygous for their respective *CYP21A2 *mutation and, therefore, clinically healthy. This insertion/duplication in exon 2 is novel (GenBank accession no. EF661662) and has not been reported previously in patients with 21-hydroxylase deficiency. This mutation was not found in 100 control subjects.

*In silico *studies were performed to understand the effect of the insertion of triple valine on the structure and function of P450c21.

### Effect on sequence conservation

**Figure 2:**
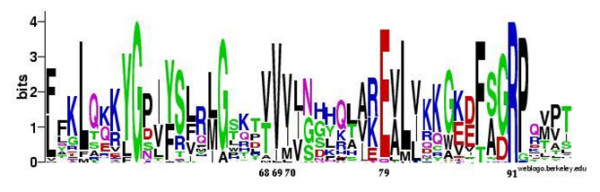
**Sequence logo depicting the conservation of residues near the insertion site**. The triple valine insertion occurs at position 71. The insertion site is flanked by many conserved residues.

The first step towards elucidating the effect of the insertion on the structure-function characteristics of the protein was to investigate the occurrence of conserved residues in the vicinity of the mutation. The sequence logo revealed the presence of two highly conserved, charged residues viz. E79 and R91 that occur downstream of the insertion site. In addition to these, hydrophobic residues occurring at 48, 51, 58, 61 63, 80, 81 and 82 positions and glycine residues occurring just before the insertion site at 56 and 64^th ^position are also conserved (Fig. 2). Hence, the mutation could cause a drastic change in the position of these conserved residues and thus lead to loss of structure and function of the protein.

### Secondary structure prediction

The secondary structures for human P450c21 were predicted using various online softwares (Table 2). Except for a few regions, there was good agreement in the predictions from the various servers (see Additional file 2). There does not seem to be any discordance with the secondary structures predicted at the site of mutation (see Additional file 2).

### Identification of suitable template

Cytochrome P4502c5 of rabbit (PDB ID: 1DT6) was identified as the best template, for modeling the structure of human P450c21, by FUGUE (Z-score of 33.12; "*certain*" with 99% confidence). As compared to the other templates, it also showed full-length sequence alignment with least number of gaps. The consensus predicted secondary structural elements of human P450c21 also aligned fairly well (see Additional file 3). There exists another experimentally elucidated structure for rabbit P4502c5 [1N6B; [44]] with a better resolution as compared to 1DT6. The secondary structure compositions of both these PDB entries are identical and the RMSD between them is 0.8 Å. Hence, 1N6B was considered as the ideal template for modeling of human P450c21. A theoretical structure of human P450c21 (PDB ID: 2GEG) has been elucidated using rabbit P4502c5 (PDB ID: 1N6B) as template [29]. This structure was thus used to represent the WT and also serve as the template for modeling the MT.

### Validation of the theoretical structures

The Verify3D analyses of theoretical models of both WT and MT revealed that most of the residues had a positive score. For low-scoring regions of the model, it was observed that the corresponding regions of the template (1N6B) too shared a low score. The structural anomalies could thus have been passed down from the template during modeling (see Additional file 4). The structures were also colour-rendered based on the Verify3D scores generated by the Colorado3D server. The major part of the structures, in all the cases, had a good score (see Additional file 5).

### Structural implications of the insertion

#### Loss of structural stability

**Figure 3:**
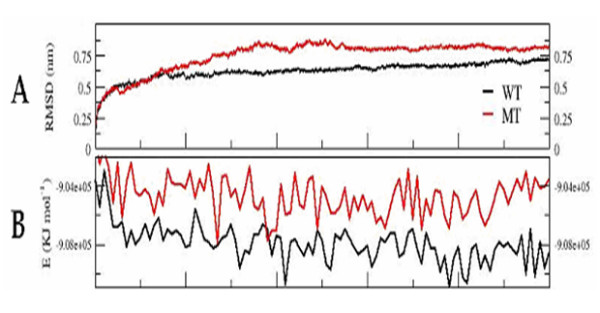
**Cα-RMSD plot of the WT and MT P450c21 structures with respect to the starting conformation during the course of the simulation (A).** The change in the potential energy of the WT and MT structures as a function of time. (B). WT and MT plots are rendered in black and red respectively. The figure was prepared using XMGRACE (38).

**Figure 4:**
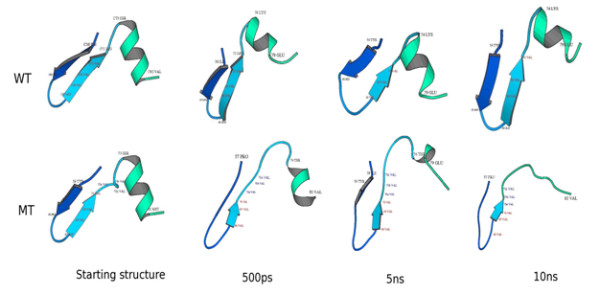
**Snapshots of the conformation of human P450c21, near the insertion site (residues 57–82), during the course of simulation**. The top and bottom panels represent the WT and MT structures respectively as a function of time. The structures have been rendered using MolScript (40) and DSSP program (39) was used for acquiring the secondary structure information.

The WT and MT structures were subjected to MD simulations. The RMSD analysis of the trajectory revealed that the WT dynamics attained equilibration at around 3 ns with an RMSD of around 0.5 nm, while the MT attained equilibrium at around 6 ns with a higher RMSD (0.75 nm; Fig. 3A). In concurrence to the above observation, the potential energy of the MT remains higher through out the dynamics as compared to the WT (Fig. 3B). The loss of the secondary structures at the region near the insertion site during the course of the simulation clearly reveals a faster unfolding of the MT as compared to the WT (Fig. 4). All these observations suggest that the triple valine insertion causes an overall destabilization of the protein.

#### Loss of H-bond interactions at the vicinity of insertion

**Figure 5:**
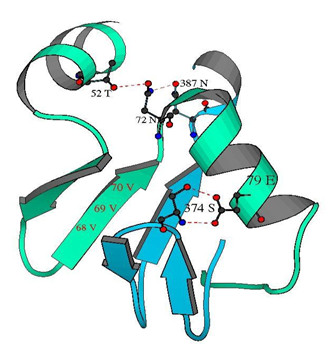
**Partial structure of human P450c21 depicting the residues whose H-bond (dotted line) interactions get affected due to mutation**. The structures in green and blue represent regions from 46–94 residues and 365–390 residues respectively. The figure has been rendered using MolScript (40).

Further examination of the atomic-level interactions at the vicinity of the insertion present in human P450c21 (WT) revealed that residues N72 and E79 participate in hydrogen bonding with distantly placed residues viz. T52, N387 and S374 (Fig. 5). Trajectory analysis revealed that the hydrogen bond interactions between E79 and S374 seem to be destroyed in the early stages of simulation for MT whereas these were retained in WT (see Additional file 6 part A). The hydrogen bonds between N72-N387 and N72-T52 were also lost rapidly for the MT (see Additional file 6 parts B and C).

### Functional implications of the insertion

#### Heme and substrate-binding

**Figure 6:**
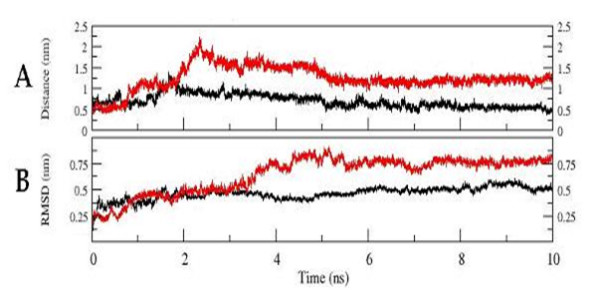
**The change in Cα distances between the residues S108 and D287, both of which are postulated to be involved in heme and substrate binding, as a function of time**. (A). Change in the CαRMSD plot of the hydrophobic patches with respect to the starting structure for WT and MT during the course of the simulation (B). WT and MT plots are rendered in black and red respectively. The figure was prepared using XMGRACE (38).

Docking and homology studies of cytochrome P450s have helped in identification of putative heme and substrate-binding residues. The residues S108 and D287 have thus been implicated in heme and substrate-binding [29]. These residues were seen to be interacting with each other in P450c21. The change in distance between the Cα atoms of these residues was monitored for the trajectories as a function of time for both the WT and MT structures (Fig. 6A). The distance between these two residues in the native WT and MT structures is 0.6 nm. It has to be noted here that the starting structures for simulations of WT and MT were identical except for the triple valine insertion and the residues S108 and D287 were distantly placed from the insertion site. In WT dynamics the distance between these two residues was maintained at around 0.6 nm, while for the MT the distance initially increased to around 2.25 nm and later stabilizes at around 1.25 nm (Fig. 6A).

#### Hydrophobic interactions

Based on Optimal Docking Area method, three segments corresponding to residues 30–42, 63–66 and 211–219 of human P450c21 were found to form a surface exposed hydrophobic patch [29]. It was hypothesized that these residues would have to participate in hydrophobic interactions to minimize the destabilizing effect caused by the surface exposed hydrophobic region. Additionally, they are positioned adjacent to the N-terminal transmembrane region of the protein. Hence these hydrophobic residues were suggested to be probably involved in binding with the ER membrane [29]. These residues were considered as a cluster and the RMS deviation of the Cα atoms of this cluster with respect to the starting conformation was compared for the WT and MT structures during the course of simulation. It was observed that the RMS deviation increased as a function of time for the MT as compared to the WT (Fig. 6B).

## Discussion

CAH is found in wide range of clinical severity ranging from subtle hormone imbalance in adults to severe life threatening salt wastage in newborns. Detection of underlying mutations in *CYP21A2 *gene encoding steroid 21-hydroxylase enzyme is helpful both for confirmation of diagnosis and management of CAH patients. Different kinds of mutations result in different degrees of enzymatic impairment of P450c21, which result in varied phenotypes of CAH patients. Although, a large number of novel mutations have been reported in *CYP21A2 *gene over the past few years and these mutations have continued expanding worldwide, large insertion/duplication mutations are uncommon. A duplication of 16 bases (CCTGGATGACACGGTC) at codons 393–397 of exon 9 [45] and a large duplication of 111 bases from codons 21 to 57 inserted at codon 58 in exon 1 of the *CYP21A2 *gene have been reported [46]. Once a novel mutation is encountered, it is necessary to carry out the functional studies to establish genotype – phenotype correlation so that the prognostic evaluation can be made for the proper management of the patient.

A large number of mutations detected in *CYP21A2 *gene have been characterized to prove their clinical relevance and impact on P450c21 protein. For characterization of mutations, functional studies have been carried out by site directed mutagenesis followed by *in vitro *expression of the mutant protein in transiently transfected mammalian cells. The residual enzyme activity is then measured towards both natural substrates (17-OHP and progesterone) and compared with the WT protein. Percentage of enzyme activity is correlated with the clinical phenotype and accordingly the mutation is classified as SV, SW or NC type [10,47-50]. However such studies are laborious, time consuming and in some mutant proteins, the biochemical and biophysical evaluation is not possible by *in vitro *studies. In such cases, *in silico *studies can provide additional clinically useful information that could not be possible by examining the patients [29]. Molecular modeling has been used to study the putative effects of steroid 21-hydroxylase gene mutations. Structural features deduced from the models were in good correlation with clinical severity of P450c21 mutants, which shows the applicability of a modeling approach in assessment of new P450c21 mutations [29,51-54].

In this particular case, the 9-bp insertion/duplication did not result in frameshift as expected from insertions/duplications in general; instead it led to insertion of three valine residues between V70 and L71. Since this insertion mutation does not exist in pseudogene, gene conversion may not be the cause of this mutation. This duplication might have been generated by intergenic recombination during meiosis. Theoretically, this insertion could lead to absence of residual 21-hydroxylase activity resulting in the severe SW phenotype. Given the fact that CAH is an autosomal recessive disorder, the clinical severity reflects the milder mutation present in the patient. Hence SV phenotype of our patient that reflected the mild mutation I172N present on one allele, could not give additional information about the possible structural perturbation caused by the novel mutation present on the other allele. In such compound heterozygous cases with a mild known mutation on one allele and a novel mutation on other allele, it is difficult to classify the mutation on the basis of clinical phenotype.

In the present study, molecular modeling and MD simulations were carried out to analyze the structural consequences of this insertion in P450c21 and to better understand the molecular pathology of CAH in the proband.

The structure of human P450c21 has not been experimentally elucidated. However, a theoretical model for it is available in the Protein Data Bank (PDB ID: 2GEG; [29]). The accuracy of this theoretical model was ascertained using structure prediction and evaluation tools (see Additional files 2, 3, 4, 5). Subsequently, using the WT as the template, the structure of the MT was modeled.

Both the WT and MT structures were subjected to MD simulations for comparison of their structural stabilities. The trajectory analyses were carried out to understand i) the effect on the structural stability and ii) plausible functional implications of the insertion.

Studies on the unfolding dynamics of the WT and MT revealed that, although the two shared an identical starting structure – except for the site of insertion, the MT exhibits faster unfolding and is less stable (Figs. 3A–B). It was also observed that the secondary structures are gradually lost at the site of insertion during the course of simulation in the MT (Fig. 4). The observations indicate that the insertion seems to have a profound effect on reducing the intrinsic stability of human P450c21.

In the absence of experimental data, the interactions and stability of the regions of the protein predicted to be harboring functional importance were selected for trajectory analyses. Based on docking and homology studies of cytochrome P450s, residues S108 and D287 have been implicated in both heme and substrate-binding [29]. Docking and structural studies have also helped in identification of hydrophobic patches in human P450c21, which are suggested to be involved in ER membrane binding [29]. The results of the present simulation studies revealed that the Cα distance between the residues S108 and D287 increase drastically in the MT while it remains the same in the WT (Fig. 3C). These residues are placed far away from the site of insertion and yet their interactions seem to be affected in the mutant. This indicates that the insertion of triple valine seems to have caused not only a local structural perturbation but also a drastic disturbance in the structural stability of protein. Similarly, in the case of the hydrophobic regions implicated in ER membrane binding, the RMSD observed during the course of simulation suggested that this region is highly unstable in the MT as compared to the WT (Fig. 3D).

The sequence logo reveals that the insertion occurs in a region flanked by the presence of many conserved residues (Fig. 2). This observation, along with the results of the trajectory analyses, strongly suggests that the mutation could lead to a loss of the structure and function of human P450c21.

## Conclusion

Analysis of *CYP21A2 *gene in an Indian child with classical CAH, revealed a novel 9 base pair TGTGGTGGT insertion at nucleotide position 306 in exon 2. This insertion resulted in a triple valine insertion between V70 and L71 of P450c21. MD simulations revealed that this insertion seems to cause an overall destabilization of the structure. Although the insertion does not occur in the immediate vicinity of the postulated heme and substrate binding residues; trajectory analyses reveal that their interactions seem to get disrupted in the MT. These observations indicate that the insertion could result in SW phenotype had it been present in homozygous state. We emphasise this mutation should be added to the panel of mutations to be screened in Indian population.

## Abbreviations

17-OHP: 17 hydroxyprogesterone; ER: Endoplasmic reticulum; GROMACS: Groningen Machine for Chemical Simulation; *hLHR*: Human Luteinizing Hormone Receptor; LINCS: Linear Constraint Solver.

## Competing interests

The authors declare that they have no competing interests.

## Authors' contributions

SD conceived and designed the study, did molecular analysis, interpreted the data and drafted the manuscript. SIT did bioinformatics studies, interpreted the data and participated in drafting the manuscript. MA participated in the *in silico *analyses and their interpretations. RRV was involved with the management of the patient. CS performed the DNA sequencing. AM participated in the design of the study and critically reviewed the draft. All authors read and approved the final manuscript.

## Supplementary Material

Additional file 1 **Fig. S1. SeqScape view.** (A) Reference sequence of *CYP21A2 *gene. (Higashi et al 1986). (B) Consensus sequence generated from forward and reverse strand. Highlighted region in grey shows insertion of 9 base pair. The vertical bar indicates the site of insertion. (C) Forward sequence of exon 2 of *CYP21A2 *gene. (D) Reverse sequence of exon 2 of *CYP21A2 *gene. (E) Results of SeqScape showing heterozygous insertion of 9 bases at position 245 of the aligned sequence. Missense substitution at position 53 and 73 are detected by the software due to noisy peaks. Click here for file

Additional file 2 **Fig. S2. Alignment of the secondary structures of human P450c21 model with the secondary structures predicted using SYMPRED, JPRED and GeneSilico metaserver**. The secondary structures are represented as black and blue highlights for helices (H) and strands (E) respectively. The arrow indicates the site of mutation. The red box highlights the discordant predictions in the secondary structure. Click here for file

Additional file 3 **Fig. S3. Sequence alignment of P4502c5 of rabbit (1DT6/1N6B) with human P450c21 generated by FUGUE server, along with their secondary structure information**. The human P450c21 secondary structure is predicted using the online servers (Table 2). If two of the three servers have agreement for a secondary structure for a residue, then that secondary structure is taken as the consensus for that residue. The arrow indicates the site of mutation. The consensus predicted secondary structure for human P450c21 is represented in the last row. The helical and strand residues are depicted in red and blue respectively. Click here for file

Additional file 4 **Fig. S4. Verify3D plot of the theoretical models of human P450c21 (WT and MT) and template structure (rabbit P4502c5; 1N6B)**. Figure containing Verify3D plot of the theoretical models of human P450c21 (WT and MT) and template structure (rabbit P4502c5; 1N6B). Click here for file

Additional file 5 **Fig. S5. Structures rendered based on the Verify3D scores generated by Colorado3D**. The region of triple valine insertion is highlighted by a box and the site of insertion is depicted by a pink star in MT. Blue and red colours indicate the best and worst scoring regions respectively. WT represents theoretical structure for human P450c21 (PDB ID: 2GEG) and the template denotes rabbit P4502c5 (PDB ID: 1N6B). Click here for file

Additional file 6 **Figure S6. The number of H-bonds between residues (A) E79-S374 (B) N72-N387 and (C) N72-T52, in WT and MT structures of human P450c21 as a function of time**. The above interactions are absent in the MT throughout the course of simulation. The figure was prepared using XMGRACE (38). Click here for file

## References

[B1] Morel Y, Miller WL (1991). Clinical and molecular genetics of congenital adrenal hyperplasia due to 21-hydroxylase deficiency. Adv Hum Genet.

[B2] White PC, Speiser PW (2000). Congenital adrenal hyperplasia due to 21-hydroxylase deficiency. Endocr Rev.

[B3] Mornet E, Crété P, Kuttenn F, Raux-Demay MC, Boue J, White PC, Boue A (1991). Distribution of deletions and seven point mutations on CYP21B genes in three clinical forms of steroid 21-hydroxylase deficiency. Am J Hum Genet.

[B4] Speiser PW, Dupont J, Zhu D, Serrat J, Buegeleisen M, Tusie-Luna MT, Lesser M, New MI, White PC (1992). Disease expression and molecular genotype in congenital adrenal hyperplasia due to 21-hydroxylase deficiency. J Clin Invest.

[B5] Wedell A, Thilén A, Ritzén EM, Stengler B, Luthman H (1994). Mutational spectrum of the steroid 21-hydroxylase gene in Sweden: implications for genetic diagnosis and association with disease manifestation. J Clin Endocrinol Metab.

[B6] Wilson RC, Wei JQ, Cheng KC, Mercado AB, New MI (1995). Rapid deoxyribonucleic acid analysis by allele-specific polymerase chain reaction for detection of mutations in the steroid 21-hydroxylase gene. J Clin Endocrinol Metab.

[B7] Bachega TAAS, Billerbeck AEC, Madureira G, Marcondes JAM, Longui CA, Leite MV, Arnhold IJP, Mendonca BB (1998). Molecular genotyping in Brazilian patients with the classical and nonclassical forms of 21-hydroxylase deficiency. J Clin Endocrinol Metab.

[B8] Yang Z, Mendoza AR, Welch TR, Zipf WB, Yu CY (1999). Modular variations of the human major histocompatibility complex class III genes for serine/threonine kinase RP, complement component C4, steroid 21-hydroxylase CYP21, and tenascin TNX (the RCCX module). A mechanism for gene deletions and disease associations. J Biol Chem.

[B9] Higashi Y, Tanae A, Inoue H, Fujii-Kuriyama Y (1998). Evidence for frequent gene conversion in the steroid 21-hydroxylase (P450c21) gene: Implications for steroid 21-hydroxylase deficiency. Am J Hum Genet.

[B10] Database of CYP21A2 by human Cytochrome P450 (CYP) Allele Nomenclature Committee. http://www.imm.ki.se/CYPalleles/cyp21.htm.

[B11] Wedell A, Luthman H (1993). Steroid 21-hydroxylase (P450c21): a new allele and spread of mutations through the pseudogene. Hum Genet.

[B12] Billerbeck AE, Bachega TA, Frazatto ET, Nishi MY, Goldberg AC, Marin ML, Madureira G, Monte O, Arnhold IJ, Mendonca BB (1999). A novel missense mutation, G424S, in Brazilian patients with 21-hydroxylase deficiency. J Clin Endocrinol Metab.

[B13] Barbaro M, Lajic S, Baldazzi L, Balsamo A, Pirazzoli P, Cicognani A, Wedell A, Cacciari E (2004). Functional analysis of two recurrent aminoacid substitution in the CYP21 gene from Italian patients with congenital adrenal hyperplasia. J Clin Endocrinol Metab.

[B14] Zhou R, Eleftheriou M, Royyuru AK, Berne BJ (2007). Destruction of long-range interactions by a single mutation in lysozyme. Proc Natl Acad Sci USA.

[B15] Latronico AC, Shinozaki H, Guerra G, Pereira MA, Lemos Marini SH, Baptista MT, Arnhold IJ, Fanelli F, Mendonca BB, Segaloff DL (2000). Gonadotropin-independent precocious puberty due to luteinizing hormone receptor mutations in Brazilian boys: a novel constitutively activating mutation in the first transmembrane helix. J Clin Endocrinol Metab.

[B16] Seibold SA, Cukier RI (2007). A molecular dynamics study comparing a wild-type with a multiple drug resistant HIV protease: differences in flap and aspartate 25 cavity dimensions. Proteins.

[B17] Cuesta-Lopez S, Falo F, Sancho J (2007). Computational diagnosis of protein conformational diseases: short molecular dynamics simulations reveal a fast unfolding of r-LDL mutants that cause familial hypercholesterolemia. Proteins.

[B18] Hamza A, Cho H, Tai HH, Zhan CG (2005). Molecular dynamics simulation of cocaine binding with human butyrylcholinesterase and its mutants. J Phys Chem B.

[B19] Cheng Q, Benson DR, Rivera M, Kuczera K (2006). Influence of point mutations on the flexibility of cytochrome b_5_: molecular dynamics simulations of holoproteins. Biopolymers.

[B20] Achary MS, Reddy AB, Chakrabarti S, Panicker SG, Mandal AK, Ahmed N, Balasubramanian D, Hasnain SE, Nagarajaram HA (2006). Disease-causing mutations in proteins: structural analysis of the CYP1b1 mutations causing primary congenital glaucoma in humans. Biophys J.

[B21] Ceruso MA, Periole X, Weinstein H (2004). Molecular dynamics simulations of transducin: interdomain and front to back communication in activation and nucleotide exchange. J Mol Biol.

[B22] Menchise V, Corbier C, Didierjean C, Saviano M, Benedetti E, Jacquot JP, Aubry A (2001). Crystal structure of the wild-type and D30A mutant thioredoxin h of Chlamydomonas reinhardtii and implications for the catalytic mechanism. Biochem J.

[B23] Wedell A, Luthman H (1993). Steroid 21-hydroxylase deficiency: Two additional mutations in salt-wasting disease and rapid screening of disease causing mutations. Hum Mol Genet.

[B24] Higashi Y, Yoshioka H, Yamane M, Gotoh O, Fujii-Kuriyama Y (1986). Complete nucleotide sequence of two steroid 21-hydroxylase genes tandemly arranged in human chromosome: a pseudogene and a genuine gene. Proc Natl Acad Sci USA.

[B25] Kurowski MA, Bujnicki JM (2003). GeneSilico protein structure prediction meta-server. Nucleic Acids Res.

[B26] Cuff JA, Clamp ME, Siddiqui AS, Finlay M, Barton GJ (1998). Jpred: A Consensus Secondary Structure Prediction Server. Bioinformatics.

[B27] Simossis VA, Heringa J (2004). Optimally segmented consensus secondary structure prediction. Bioinformatics.

[B28] Shi J, Blundell TL, Mizuguchi K (2001). FUGUE: sequence-structure homology recognition using environment-specific substitution tables and structure-dependent gap penalties. J Mol Biol.

[B29] Robins T, Carlsson J, Sunnerhagen M, Wedell A, Persson B (2006). Molecular model of human CYP21 based on mammalian CYP2C5: Structural features correlate with clinical severity of mutations causing congenital adrenal hyperplasia. Mol Endocrinol.

[B30] Lüthy R, Bowie JU, Eisenberg D (1992). Assessment of protein models with three-dimensional profiles. Nature.

[B31] Sasin JM, Bujnicki JM (2004). COLORADO3D, a web server for the visual analysis of protein structures. Nucleic Acids Res.

[B32] Van der Spoel D, Lindahl E, Hess B, Groenhof G, Mark AE, Berendsen HJC (2005). GROMACS: Fast, Flexible and Free. J Comput Chem.

[B33] Darden T, York D, Pedersen L (1993). Particle mesh Ewald: An N-log(N) method for Ewald sums in large systems. J Chem Phys.

[B34] Berendsen HJC, Postma JPM, van Gunsteren WF, Hermans J, Pullman B (1981). Intermolecular Forces. Interaction models for water in relation to protein hydration.

[B35] Lin FH, Graham LA, Campbell RL, Davies PL (2007). Structural modeling of snow flea antifreeze protein. Biophy J.

[B36] Louie TM, Yang H, Karnchanaphanurach P, Xie XS, Xun L (2002). FAD is a preferred substrate and an inhibitor of Escherichia coli general NAD(P)H: flavin oxidoreductase. J Biol Chem.

[B37] Hess B, Bekker H, Berendsen HJC, Fraaije JGEM (1997). LINCS: A linear constraint solver for molecular simulations. J Comput Chem.

[B38] Turner PJ (2005). XMGRACE, Version 5.1.19.

[B39] Kabsch W, Sander C (1983). Dictionary of protein secondary structure: pattern recognition of hydrogen-bonded and geometrical features. Biopolymers.

[B40] Kraulis PJ (1991). *MOLSCRIPT*: a program to produce both detailed and schematic plots of protein structures. J Appl Crystallogr.

[B41] Crooks GE, Hon G, Chandonia JM, Brenner SE (2004). WebLogo: A sequence logo generator. Genome Res.

[B42] Altschul SF, Madden TL, Schäffer AA, Zhang J, Zhang Z, Miller W, Lipman DJ (1997). Gapped BLAST and PSI-BLAST: a new generation of protein database search programs. Nucleic Acids Res.

[B43] Thompson JD, Higgins DG, Gibson TJ (1994). CLUSTAL W: improving the sensitivity of progressive multiple sequence alignment through sequence weighting, position specific gap penalties and weight matrix choice. Nucleic Acids Res.

[B44] Wester MR, Johnson EF, Marques-Soares C, Dansette PM, Mansuy D, Stout CD (2003). Structure of a substrate complex of mammalian cytochrome P450 2C5 at 2.3 A resolution: Evidence for multiple substrate binding modes. Biochemistry.

[B45] Lee HH, Chao HD, Lee YJ, Shu SG, Chao MC, Kuo JM, Chung BC (1998). Identification of four novel mutations in the CYP21 gene in congenital adrenal hyperplasia in the Chinese. Hum Genet.

[B46] Lee HH, Chang SF, Lo FS, Chao HT, Lin CY (2003). Duplication of 111 bases in exon 1 of the *CYP21 *gene is combined with deletion of *CYP21P-C4B *genes in steroid 21 hydroxylase deficiency. Mol Genet Metab.

[B47] Robins T, Bellanne-Chantelot C, Barbaro M, Cabrol S, Wedell A, Lajic S (2007). Characterization of novel missense mutations in *CYP21 *causing congenital adrenal Hyperplasia. J Mol Med.

[B48] Menassa R, Tardy V, Despert F, Bouvattier-Morel C, Brossier JP, Cartigny M, Morel Y (2008). p.H62L, a rare mutation of the CYP21 gene identified in two forms of 21-hydroxylase deficiency. J Clin Endocrinol Metab.

[B49] Soardi FC, Barbaro M, Lau IF, Lemos-Marini SHV, Baptista MTM, Guerra-Junior G, Wedell A, Lajic S, de Mello MP (2008). Inhibition of CYP21A2 Enzyme Activity Caused by Novel Missense Mutations Identified in Brazilian and Scandinavian Patients. J Clin Endocrinol Metab.

[B50] Riepe FG, Hiort O, Grötzinger J, Sippell WG, Krone N, Holterhus PM (2008). Functional and structural consequences of a novel point mutation in the CYP21A2 gene causing congenital adrenal hyperplasia: potential relevance of helix C for P450 oxidoreductase-21-hydroxylase interaction. J Clin Endocrinol Metab.

[B51] Krone N, Riepe FG, Grotzinger J, Partsch CJ, Sippell WG (2005). Functional characterization of two novel point mutations in the CYP21 gene causing simple virilizing forms of congenital adrenal hyperplasia due to 21-hydroxylase deficiency. J Clin Endocrinol Metab.

[B52] Janner M, Pandey AV, Mullis PE, Flück CE (2006). Clinical and biochemical description of a novel *CYP21A2 *gene mutation 962_963insA using a new 3D model for the P450c21 protein. Eur J Endocrinol.

[B53] Grischuk Y, Rubtsov P, Riepe FG, Grotzinger J, Beljelarskaia S, Prassolov V, Kalintchenko N, Semitcheva T, Peterkova V, Tiulpakov A, Sippell WG, Krone N (2006). Four Novel Missense Mutations in the *CYP21A2 *Gene Detected in Russian Patients Suffering from the Classical Form of Congenital Adrenal Hyperplasia: Identification, Functional Characterization, and Structural Analysis. J Clin Endocrinol Metab.

[B54] Baradaran-Heravi A, Vakili R, Robins T, Carlsson J, Ghaemi N, A'rabi A, Abbaszadegan MR (2007). Three novel *CYP21A2 *mutations and their protein modelling in patients with classical 21-hydroxylase deficiency from northeastern Iran. Clin Endocrinol.

