# Supplementary figures and images for: A novel 9-bp insertion detected in steroid 21-hydroxylase gene (CYP21A2): prediction of its structural and functional implications by computational methods
Source: J Biomed Sci. 2009 Jan 8;16(1):3. doi: 10.1186/1423-0127-16-3 (PMC2653521; doi:10.1186/1423-0127-16-3)

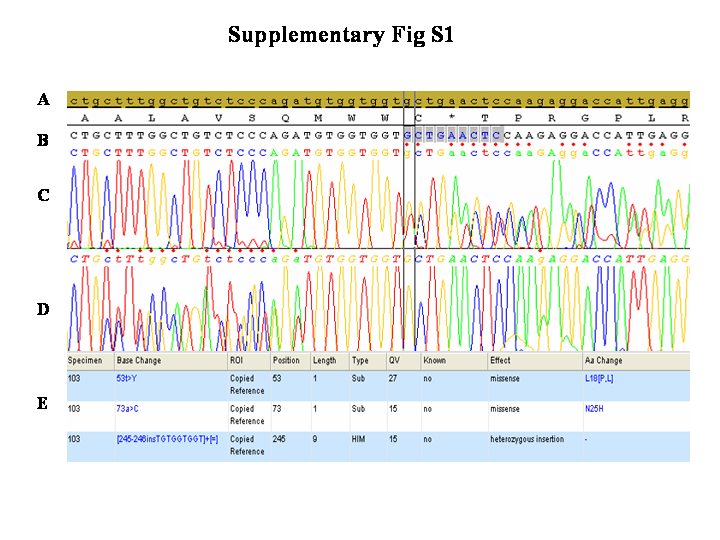

Supplement: Additional file 1 — Fig. S1. SeqScape view. (A) Reference sequence of CYP21A2 gene. (Higashi et al 1986). (B) Consensus sequence generated from forward and reverse strand. Highlighted region in grey shows insertion of 9 base pair. The vertical bar indicates the site of insertion. (C) Forward sequence of exon 2 of CYP21A2 gene. (D) Reverse sequence of exon 2 of CYP21A2 gene. (E) Results of SeqScape showing heterozygous insertion of 9 bases at position 245 of the aligned sequence. Missense substitution at position 53 and 73 are detected by the software due to noisy peaks. [file 1423-0127-16-3-S1.tiff]

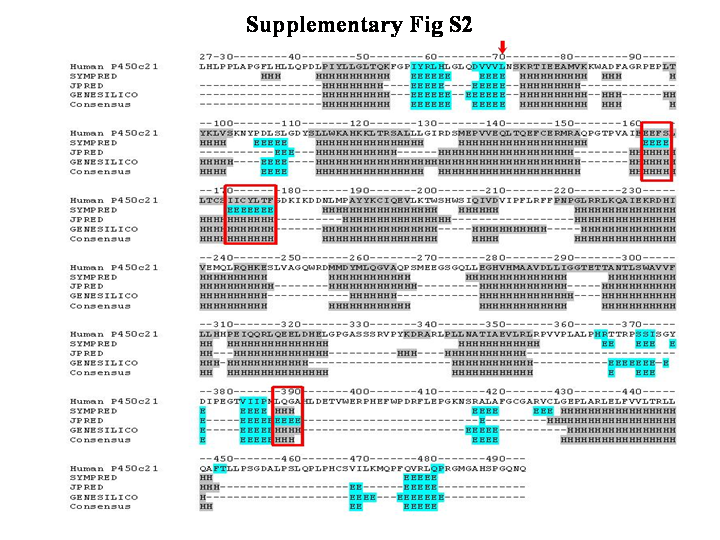

Supplement: Additional file 2 — Fig. S2. Alignment of the secondary structures of human P450c21 model with the secondary structures predicted using SYMPRED, JPRED and GeneSilico metaserver. The secondary structures are represented as black and blue highlights for helices (H) and strands (E) respectively. The arrow indicates the site of mutation. The red box highlights the discordant predictions in the secondary structure. [file 1423-0127-16-3-S2.tiff]

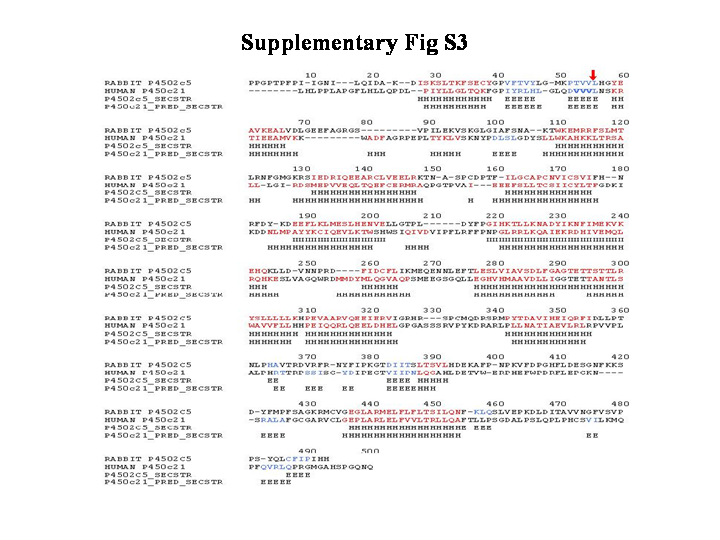

Supplement: Additional file 3 — Fig. S3. Sequence alignment of P4502c5 of rabbit (1DT6/1N6B) with human P450c21 generated by FUGUE server, along with their secondary structure information. The human P450c21 secondary structure is predicted using the online servers (Table 2). If two of the three servers have agreement for a secondary structure for a residue, then that secondary structure is taken as the consensus for that residue. The arrow indicates the site of mutation. The consensus predicted secondary structure for human P450c21 is represented in the last row. The helical and strand residues are depicted in red and blue respectively. [file 1423-0127-16-3-S3.tiff]

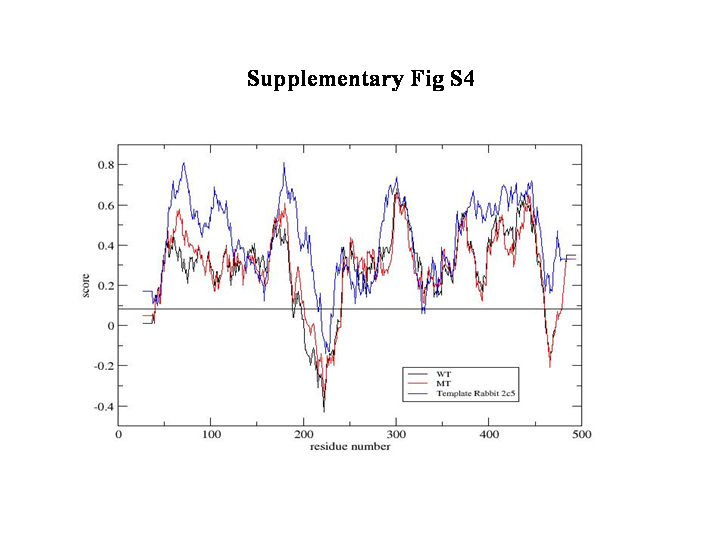

Supplement: Additional file 4 — Fig. S4. Verify3D plot of the theoretical models of human P450c21 (WT and MT) and template structure (rabbit P4502c5; 1N6B). Figure containing Verify3D plot of the theoretical models of human P450c21 (WT and MT) and template structure (rabbit P4502c5; 1N6B). [file 1423-0127-16-3-S4.tiff]

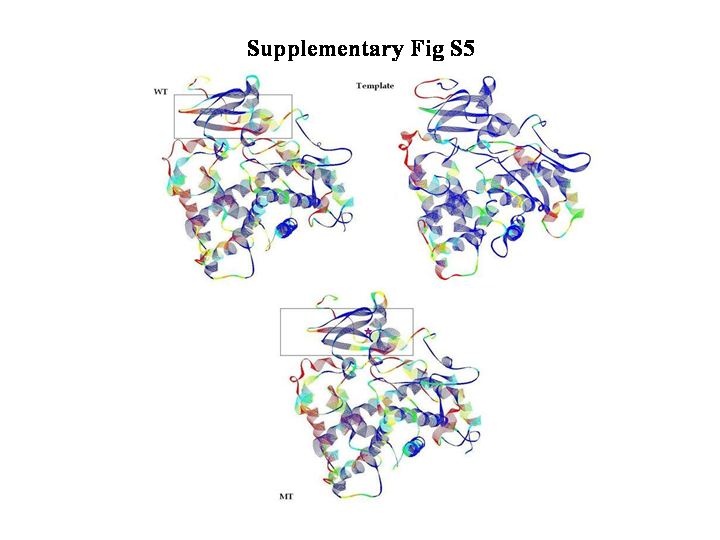

Supplement: Additional file 5 — Fig. S5. Structures rendered based on the Verify3D scores generated by Colorado3D. The region of triple valine insertion is highlighted by a box and the site of insertion is depicted by a pink star in MT. Blue and red colours indicate the best and worst scoring regions respectively. WT represents theoretical structure for human P450c21 (PDB ID: 2GEG) and the template denotes rabbit P4502c5 (PDB ID: 1N6B). [file 1423-0127-16-3-S5.tiff]

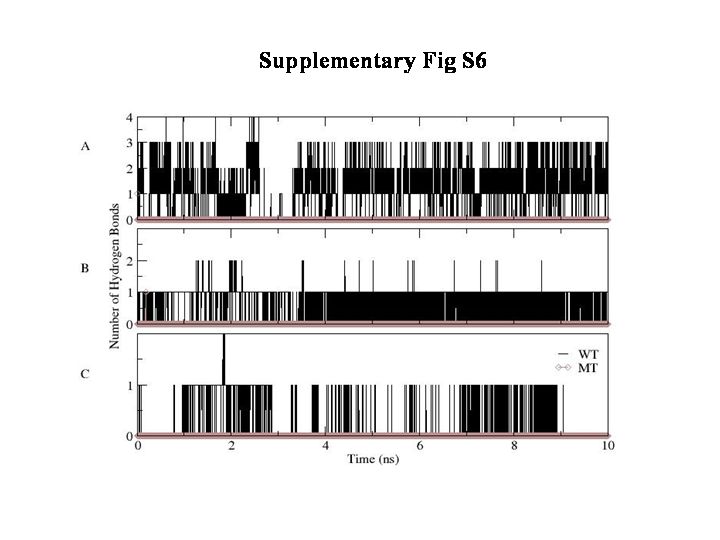

Supplement: Additional file 6 — Figure S6. The number of H-bonds between residues (A) E79-S374 (B) N72-N387 and (C) N72-T52, in WT and MT structures of human P450c21 as a function of time. The above interactions are absent in the MT throughout the course of simulation. The figure was prepared using XMGRACE (38). [file 1423-0127-16-3-S6.tiff]
